# Bioconverted *Orostachys japonicas* Extracts Suppress Angiogenic Activity of Ms-1 Endothelial Cells

**DOI:** 10.3390/ijms18122615

**Published:** 2017-12-05

**Authors:** Seul Gi Lee, Jin Soo Kim, Han-Saem Lee, Yu-Mi Lim, Jai-Hyun So, Dongyup Hahn, Yu Shin Ha, Ju-Ock Nam

**Affiliations:** 1 Department of Food Science and Biotechnology, Kyungpook National University, Daegu 41566, Korea; lsg100479@naver.com (S.G.L.); 1211pepero@naver.com (J.S.K.); 2 National Development Institute of Korean Medicine, 94, Hwarang-ro, Gyeongsan, Gyeongsangbuk-do 712-260, Korea; hansaem35@nikom.or.kr (H.-S.L.); yum120@nikom.or.kr (Y.-M.L.); dukeny@hanmail.net (J.-H.S.); 3 Institute of Agricultural Science and Technology, Kyungpook National University, Daegu 41404, Korea; dohahn@knu.ac.kr; 4 Department of Bio Industrial Machinery Engineering, Kyungpook National University, Daegu 41566, Korea

**Keywords:** anti-angiogenic, bioconversion, Ms-1 endothelial cells, *Orostachys japonicus* A. Berger, Wa-song

## Abstract

*Orostachys japonicus* A. Berger (), known as Wa-song in Korea, has been reported to exert various biological effects, such as anti-tumor, anti-oxidant, and anti-febrile effects. However, the anti-angiogenic effects of *O.* *japonicus* extracts remain to be investigated. In the present study, we demonstrated the anti-angiogenic effects of bioconverted *O. japonicus* extract (BOE) in Ms-1 mouse endothelial cells and compared them with the bioactivities of *O. japonicus* extract (OE). BOE, but not OE, were found to exert anti-angiogenic effects, including inhibition of cell migration, cell adhesion, tube formation of Ms-1 cells, and blood vessel formation of matrigel plug assay in vivo. Furthermore, protein levels of phosphorylated Src kinase were lower in BOE-treated cells than in OE-treated cells. Treatment with OE or BOE did not influence cell viability during the experimental period. Bioconverted extract of *O.* *japonicus* have anti-angiogenic effects in vitro and vivo, but non-bioconverted extract do not. We suggest that these observed anti-angiogenic effects are caused by the changes in the composition of bioactive compounds in the extracts as a result of biological conversion.

## 1. Introduction

*Orostachys japonicus* A. Berger (*O*. *japonicus*), known as rock pine (in English) or as Wa-song (local name in Korea), has been used as folk medicine to treat gastric ulcer or gastric cancer disease in Korea [1,2]. *O. japonicas* extracts contain triterpenoid, sterol, aromatic acid, and flavonoids such as kaempferol and quercetin [1,3]. Many studies have investigated the various biological effects of *O. japonicus* extracts, such as anti-tumor, anti-oxidant, and anti-febrile effects [2,4].

Bioconversion is a process that enhances the therapeutic effects of herbal medicine by changing the secondary metabolite composition and altering the mechanisms that are responsible for biological activity [5,6]. Moreover, bioconverted extracts are more likely to contain novel or abundant bioactive substances than normal herbal extracts [7].

Angiogenesis plays an important role in many physiological and pathological processes, such as heart disease, inflammation, and cancer [8,9]. Tumor growth and metastasis require the formation of new blood vessels. Thus, inhibition of the angiogenic process represents an effective strategy for cancer treatment [10,11].

However, the effects of *O. japonicus* extracts on angiogenesis of endothelial cells have not yet been investigated. Therefore, we aimed to evaluate the effects of non-bioconverted *O. japonicus* extract (OE) and bioconverted *O. japonicus* extract (BOE) on angiogenesis of endothelial cells and determine the possible mechanisms of action.

## 2. Results and Discussion

### 2.1. O. japonicus Extract (OE) and Bioconverted O. japonicus Extract (BOE) Did Not Affect the Viability of Ms-1 Cells

**Figure 1:**
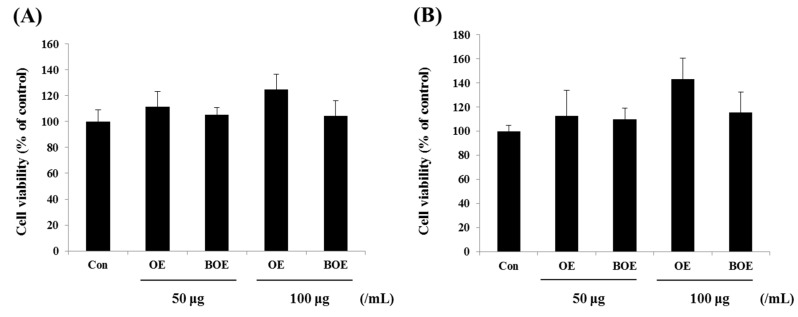
Effects of *Orostachys japonicus* extract (OE) and bioconverted *O. japonicus* extract (BOE) on Ms-1 cell viability. Cell viabilities of Ms-1 cells were examined using the MTT assay at 24 h (**A**) or 48 h (**B**) following OE or BOE treatment at the indicated concentrations. Bars represent mean ± SD of three independent experiments. Con, control group.

Before evaluating the anti-angiogenic effects of OE and BOE, we first determined the cytotoxicity of OE and BOE on Ms-1 endothelial cells. Cell proliferation and viability were not significantly affected by treatment with OE and BOE at concentrations ranging from 0–100 μg/mL. Prolonged treatment with OE and BOE showed similar results (Figure 1A,B). Accordingly, we used OE and BOE at a concentration range of 0–100 μg/mL for subsequent experiments.

### 2.2. BOE Suppress the Migration and Adhesion Abilities of Ms-1 Cells

**Figure 2:**
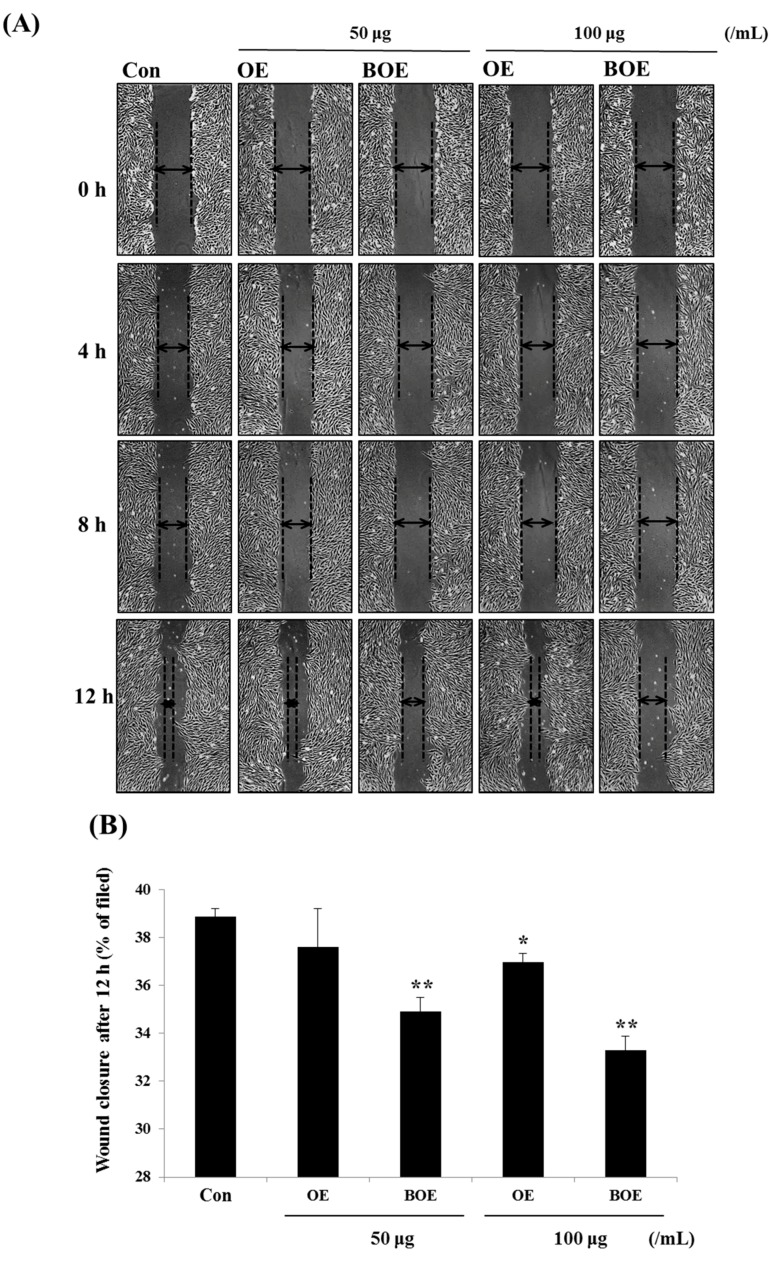

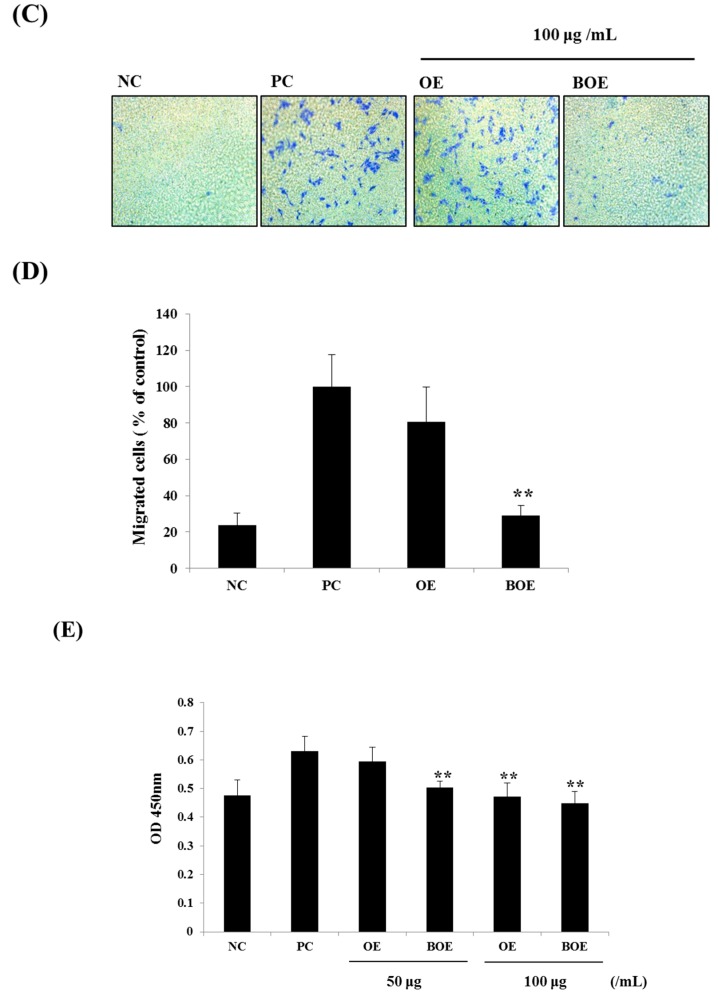
Effects of OE and BOE on cell migration and adhesion abilities of Ms-1 cells. (**A**) Cells were scratched and treated with the indicated concentrations of OE or BOE. The control (Con) was treated with the same amount of DMSO. Representative images were photographed under a microscope at 100× magnification. Dased lines indicate the wound edge on the right and left side of the scratch. (**B**) The wound closure area was quantified by ImageJ software (National Institute of Health, Bethesda, MD, USA). (**C**) Cells were pretreated with OE or BOE, and numbers of migrated cells were quantified (**D**) using ImageJ software. The cells were counted in three random parts at 200× magnification. (**E**) Cells were pre-treated with OE or BOE, and the number of adherent cells were measured using CCK-8 solution. BSA (bovine serum albumin)-coated control and FN (fibronectin)-coated control were used as negative control (NC) and positive control (PC), respectively. *p* < 0.01 (**); *p* < 0.05 (*). Bars represent mean ± SD of three independent experiments.

Cell migration is an essential process in growth, development, and wound healing and is crucial for angiogenesis [12,13]. Next, we examined the effects of OE and BOE on cell mobility. Results of the wound healing assay showed that treatment with BOE suppressed cell migration in a dose-dependent manner, as evidenced by the measured areas of wound healing and the formation of the leading edge shape by migrating cells (Figure 2A,B). Also, BOE significantly inhibited cell migration based on the results of the trans-well migration assay (Figure 2C,D). Furthermore, BOE treatment decreased the adhesion of Ms-1 cells to fibronectin (FN), whereas OE did not affect the migration and adhesion abilities of cells (Figure 2E). Although the differences were not significant, BOE more strongly inhibited cell adhesion than OE at the concentration of 100 μg/mL. These results imply that the anti-angiogenic activity of BOE was enhanced after the bioconversion process and thus do not indicate new effects.

### 2.3. BOE Inhibits Angiogenesis of Ms-1 Cells

**Figure 3:**
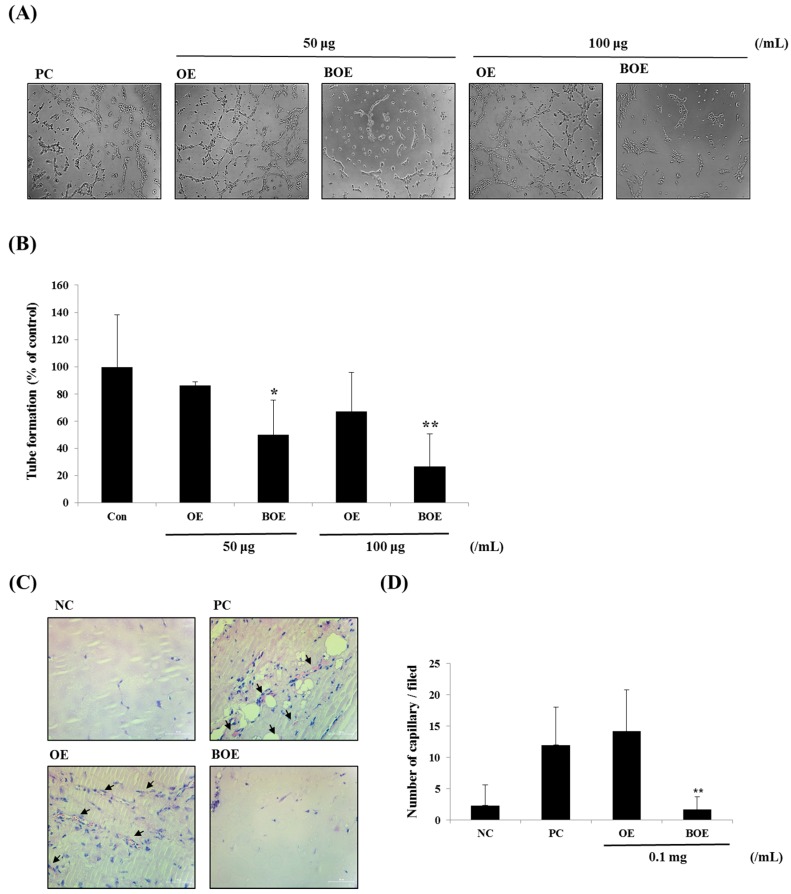
Effects of OE and BOE on tube formation of Ms-1 cells. (**A**) Cells were pretreated with OE or BOE and seeded onto plates pre-coated with matrigel. Representative images were photographed with a microscope at 200× magnification. (**B**) Tube lengths were quantified using ImageJ. (**C**) Matrigel plugs were harvested and stained with hematoxylin and eosin stain (H&E). The arrows represented infiltrating vessels. Representative images were photographed with a microscope at 400× magnification. (**D**) The number of vessels was counted. *p* < 0.01 (**); *p* < 0.05 (*). Bars represent mean ± SD.

Matrigel-induced tube formation in vitro assay is a highly reliable approach used to determine the antiangiogenic properties of drugs [14]. BOE treatment significantly disrupted the formation of capillary web structures, whereas OE treatment resulted in well-formed capillary web structures similar to those of the control group (Figure 3A). Treatment with 50 and 100 μg/mL of BOE reduced tube formation by 50.4% and 26.7%, respectively (Figure 3B). Next, we determined whether the BOE have direct anti-angiogenic ability in vivo using matrigel plug assay. OE or BOE at concentration of 0.1 mg/mL were mixed with matrigel. Only BOE significantly inhibited blood vessels infiltrated into the matrigel plug; OE did not (Figure 3C,D).

### 2.4. BOE Inhibited FAK (Focal Adhesion Kinase) and Src Activation

**Figure 4:**
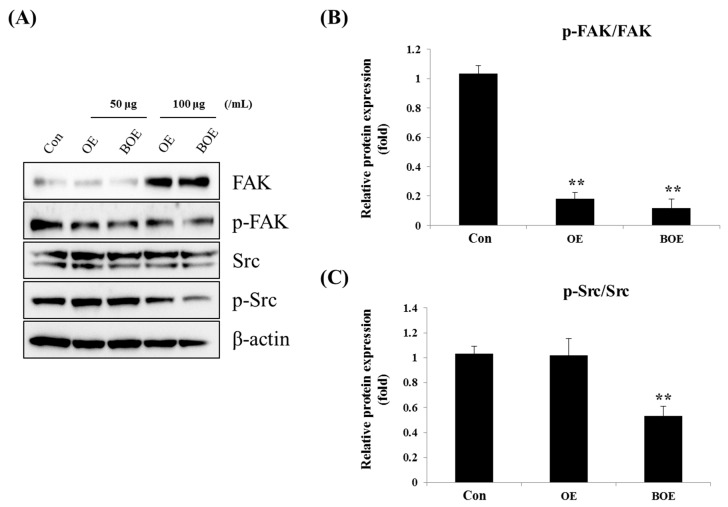
Effects of OE and BOE on the activation of FAK (focal adhesion kinase) and Src. Cells were lysed after treatment with OE or BOE for 24 h. (**A**) Protein expression of p-FAK, total FAK, p-Src, total Src, and β-actin were analyzed via western blotting. The level of phosphorylation of FAK (**B**) and Src (**C**) was measured by fusion solo detector software. Photographs are representative pictures from three independent experiments. *p* < 0.01 (**). Bars represent mean ± SD.

FAK (focal adhesion kinase) and Src signaling promote tumor angiogenesis through the regulation of endothelial cell migration and tube formation [15,16]. To verify the mechanisms responsible for the anti-angiogenic effects of BOE, we examined the effects of OE and BOE on the activation of FAK and Src. Results showed that BOE treatment significantly reduced the expression levels of phosphorylated FAK and Src in a dose-dependent manner (Figure 4A–C).

### 2.5. Effects of Bioconversion on Changing Ultra Performance Liquid Chromatography (UPLC)Patterns of O. japonicus

**Figure 5:**
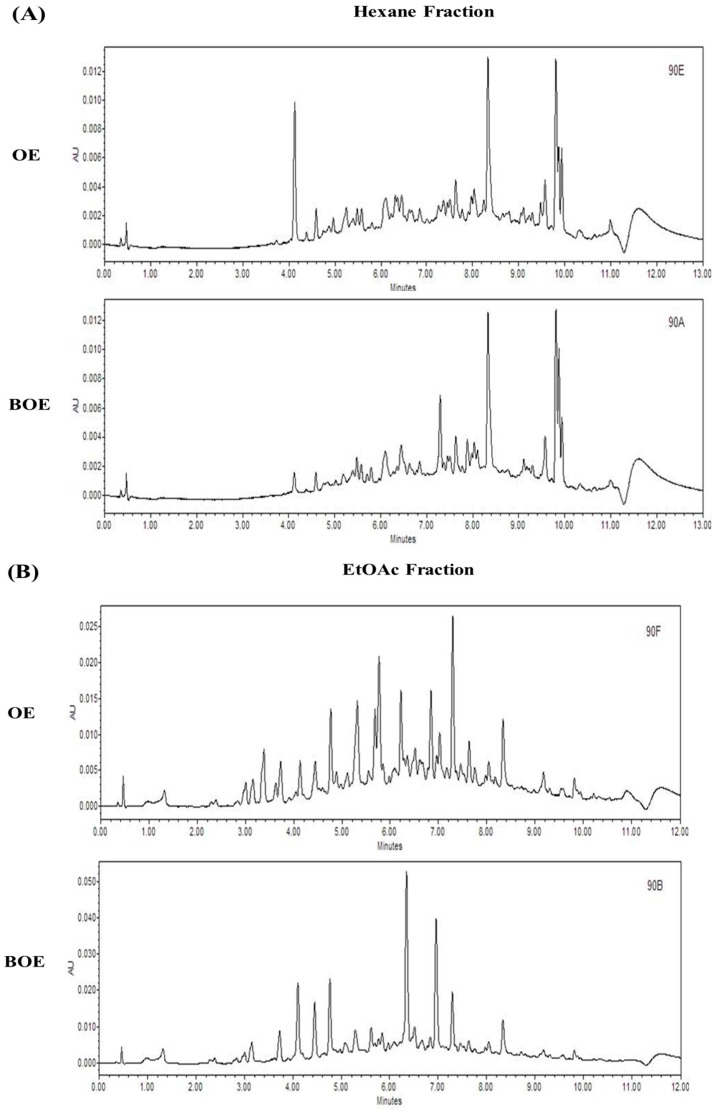

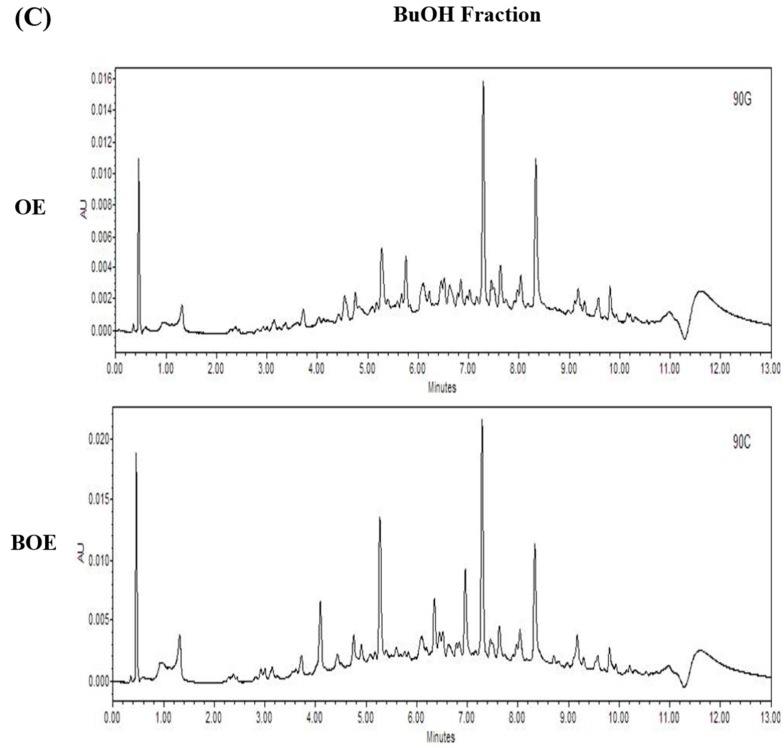
Comparison of UPLC chromatogram of *O. japonicus* extracts (OE) and bioconverted *O. japonicus* extracts (BOE). The UPLC results of hexane fraction (**A**), Ethyl acetate (EtOAc) fraction (**B**), and Butanol (BuOH) fraction (**C**) are shown.

To compare the ultra performance liquid chromatography (UPLC) patterns of OE and BOE, we performed UPLC analysis. Each fraction shows significant change in the composition of compounds by means of bioconversion (Figure 5A–C). These results suggest that bioconversion resulted in a change of compounds in the extract.

## 3. Materials and Methods

### 3.1. Sample Preparation and Cell Culture

The OE and BOE were obtained from the National Development Institute of Korean Medicine. *O. japonicus* was purchased from Oriental Medicine Market in Korea (Daegu) and was refluxed twice with 3.5 L of 70% MeOH (a sample/MeOH ratio of 1:7 (*w*/*w*)). The extract was filtered through filter paper and concentrated using a Rotary evaporator (EYELA, Tokyo, Japan).

The resulting extract was bioconverted using the enzyme isolated from the soybean paste fungi, *Aspergillus kawachii* (*A. kawachii*), according to a previous study with some modifications [17]. Briefly, extracts were resuspended in 200 mL of distilled water, and a 100 mL aliquot of the suspension was treated with 100 mL of active or inactive crude enzyme extract. The mixture was incubated with shaking at 100 rpm at 30 °C. Inactive crude enzyme prepared by autoclaving the mixture at 121 °C for 15 min served as the control.

For cell culture, Ms-1 mouse endothelial cells were obtained from Ha-Jeong Kim (Kyungpook National University) and cultured in Dulbecco’s Modified Eagle’s Medium (DMEM) high supplemented with 10% fetal bovine serum (FBS) at 37 °C with humidified air containing 5% CO_2_. DMEM and FBS were purchased from Gibco Life Technologies (Grand Island, NY, USA).

### 3.2. Preparation of A. kawachii Enzyme

The *A. kawachii* enzyme was prepared as previously described [17]. Briefly, *A. kawachii* grown in potato dextrose agar (PDA) medium (Difco Laboratories, Detroit, MI, USA) was inoculated into sterilized wheat bran and incubated at 30 °C for 3 days. The resulting mixture was suspended in sodium phosphate buffer (pH 7.0) and incubated for 18 h at 4 °C. The reaction mixture was then centrifuged, and the supernatant was used for fermentation of *O. japonicus* extracts. The supernatant provided 0.276 U/mL (1 U is defined as the enzyme activity needed to produce 1 mmol of *p*-nitrobenzene from *p*-nitrophenyl-β-d-glucopyranoside per min) β-glucosidase activity [17].

### 3.3. UPLC Analysis 

To confirm the changing compounds during bioconversion were quantified by Waters UPLC (ACQUITY Ultra Performance LC systems H class, Waters, Milford, MA, USA) with the wavelength set at 280 nm. The sample was dissolved to 0.5 mg/mL in MeOH and filtered through a 0.45 µm membrane filter and 2 µL of filtrate was analyzed. The UPLC analyses were performed using a ACQUITY UPLC CSH C18 (2.1 × 100 mm, 1.7 µm; Waters) reverse phase column and the mobile phase consisted of water (solvent A) and MeCN (solvent B) each containing 0.1% formic acid. After the sample was injected into the column, solvent B increased to 100% in 10 min, then decreased to 0% in 1 min and held at 0% for 2 min. The solvent flow rate was 0.3 mL/min.

### 3.4. MTT (3-(4,5)-Dimethylthiazo(-2-y1)-2,5-diphenytetrazolium bromide) Assay

Ms-1 cells were seeded in 96-well plates and treated with 50 or 100 μg/mL OE or BOE for 24 h. Control groups were treated with the same amounts of Dimethyl sulfoxide (DMSO). 3-(4,5)-Dimethylthiazo(-2-y1)-2,5-diphenytetrazolium bromide (MTT) solution was added to the cells, which were then incubated at 37 °C for 3 h. The solution was removed, and the precipitated formazan was dissolved with isopropyl alcohol (Duksan Pure Chemicals, Ansan, Korea). The absorbance of each sample was measured at 595 nm.

### 3.5. Adhesion Assay

The 96 well-plates were precoated with 5 μg/mL fibronectin (FN) or 2% BSA, blocked with 0.5% BSA for 1 h, and washed with PBS. Single-cell suspensions of Ms-1 cells were pretreated with OE or BOE for 30 min, seeded on coated 96-well plates, and then allowed to adhere for 30 min at 37 °C. The number of adherent cells were measured using cell counting kit-8 reagents (CCK-8, Do-jindo, Kumamoto, Japan) according to the manufacturer’s instructions. Briefly, each well was washed, CCK-8 solution was added, and the plates were incubated for 4 h at 37 °C. The absorbance of each sample was measured at 450 nm.

### 3.6. Wound Healing and Migration Assay

For the wound healing assay, Ms-1 cells were seeded in 6-well plates and incubated until post-confluence. A scratch was created using a 200 μL pipette tip, after which cells were treated with OE or BOE and cultured in DMEM-high without serum. Cells were photographed at each time point (0, 4, 8, and 12 h), and the wound closure area was measured using ImageJ software (National Institute of Health, Bethesda, MD, USA). The wound closure area was calculated using the following formula: migration rate (%) = (average wound distance − average no migration distance)/average wound distance × 100.

For the migration assay, trans-well chamber inserts (polycarbonate membrane, 8-μm pore size; Costar, MA, USA) were coated with 5 μg/mL FN or 2% BSA, blocked with 1% BSA for 30 min, and washed with PBS. Single cell suspensions of Ms-1 cells were pretreated with OE or BOE for 30 min, seeded onto the upper insert of the chamber, and allowed to migrate for 8 h at 37 °C. Inserts were then cleaned with cotton swabs, fixed with 6.0% glutaraldehyde, and stained with 0.1% crystal violet. Migrated cells were photographed and counted using ImageJ software.

### 3.7. Tube Formation Assay

Matrigel (Corning, New York, NY, USA) was added to 96-well plates (90 μL/well) and incubated for 30 min at 37 °C to allow polymerization. Ms-1 cells were pretreated with OE or BOE for 30 min, seeded, and incubated for 5 h at 37 °C. The formation of tube structures were confirmed and imaged with a microscope. Tube lengths were quantified using ImageJ software.

### 3.8. Matrigel Plug Assay

Six week-old C57BL/6 male mice were used. Matrigel (BD Bioscience, San Jose, CA, USA) was mixed with 20 U/mL heparin, 200 ng/mL VEGF (R&D system, Minneapolis, MN, USA), and with or without the OE or BOE. The matrigel mixture was injected in subcutaneous (sc) and after 3 weeks the mice were sacrificed, then, the matrigel plug was removed and processed for histologic analysis as previously described [18]. Briefly, the matrigel plugs were embedded sectioned, and hematoxylin and eosin stain (H&E) stained. The number of erythrocyte-filled blood vessels was counted under a light microscope (400×). Each group consisted of five or six matrigel plugs. The animal study and protocols were approved by the Institutional Animal Care Committee of Kyungpook National University (approval number: KNU 2017-0120; 6 September 2017).

### 3.9. Western Blotting

Cells were lysed in radioimmunoprecipitation assay lysis buffer. Total proteins were separated via 10–15% sulfate-polyacrylamide gel electrophoresis (SDS-PAGE), transferred onto nitrocellulose membranes, blocked in 5% non-fat skim milk in TBST (10 mM tris, 150 mM NaCl, and 0.05% Tween 20), and subsequently incubated with the following primary antibodies: phospho-FAK, FAK (Cell Signaling Technology, Beverly, MA, USA), phospho-Src, Src, and β-actin (Santa Cruz Biotechnology, Santa Cruz, CA, USA). Relative expression level of proteins was quantified using the Fusion Solo Detector (Vilber Lourmat, Marne-la-Vallée, France).

### 3.10. Statistical Analysis 

Statistical analyses were performed using one-way ANOVA for experiments with at least three biological replicates. *p* < 0.05 was considered statistically significant.

## 4. Conclusions

We demonstrated that bioconverted extract of *O. japonicus* inhibits the migration, adhesion, and tube formation of endothelial cells even at concentrations where non-bioconverted extract is ineffective. These results suggest that bioconverted extract of *O. japonicus* greatly enhance anti-angiogenic effects. Thus, we propose that bioconverted extract of *O. japonicus* can be developed as a more potent angiogenic inhibitor to protect tumor growth.
